# The End of Compulsory Gender Verification: Is It Progress for Inclusion of Women in Sports?

**DOI:** 10.1007/s10508-021-02073-x

**Published:** 2021-09-07

**Authors:** Jonathan Ospina-Betancurt, Eric Vilain, María José Martinez-Patiño

**Affiliations:** 1 Departamento de Ciencias del Deporte, Faculty of Sport Science, Universidad Europea de Madrid, Madrid, Spain; 2 Center for Genetic Medicine Research, Children’s National Research Institute, Children’s National Hospital, 111 Michigan Ave. NW, Washington, DC 20010 USA; 3 Department of Genomics and Precision Medicine, George Washington University School of Medicine and Health Sciences, Washington, DC USA; 4 Facultad de Ciencias de la Educación y del Deporte, University of Vigo, Vigo, Spain

**Keywords:** Women’s athletics, Caster Semenya, International Association of Athletics Federations, Court of Arbitration for Sport, Gender policies, Disorders of sex development

## Abstract

Recently, the so-called Semenya case has brought the problem of gender in sports competitions back into the spotlight. But the fact is that it is not a unique case; rather, it seems a recurrent and inconclusive problem in the history of sports. In this context, the Spanish athlete Martínez-Patiño is an important figure in the history of sport and gender verification, as well as the Indian sprinter Dutee Chand. Martínez-Patiño’s story thus serves as an important case study of the gender-based anxieties that hampered women’s advancement in track and field. Martínez-Patiño’s experience in Spanish athletics demonstrates the difficulties women faced when attempting to compete in track and field, both in Spain and internationally. Moreover, her experience with gender policies shows the inadequacies of the chromosomal check as a sex marker, as well as the harms caused by the technique. Finally, Martínez-Patiño’s protest of the International Association of Athletics Federations’ policy started to dismantle compulsory sex verification used as a criterion for gender eligibility. The publicity surrounding her case pushed the track and field federation to abandon mandatory, on-site testing in 1992. Seven years later, the International Olympic Committee also dropped its compulsory control. Martínez-Patiño became the face of the fight against sex/gender verification in sport and helped dismantle the practice. The case of Martinez-Patiño remains in the collective memory of elite sports and serves as an argument for national and international sporting institutions to reconsider discriminating policies in the context of progress being made for women’s rights.

## Introduction

In 1983, the Spanish athlete Maria José Martínez-Patiño travelled to Helsinki, Finland, to compete in the World Championship in Athletics. In the words of the young athlete, “One of the best moments in my athletic career was when they informed me that I was selected to participate in the World Championship… I had trained hard for this competition and it was my opportunity to show what I could do.” Before she could demonstrate her athleticism, though, Martínez-Patiño had to undergo the requisite gender verification, an exam that used a buccal smear test to check female athletes’ chromosomes. At that time, the International Association of Athletics Federations (IAAF) required the control for all women who competed in local, continental, or world championships, arguing that a person’s chromosomal composition determined her sex (Ferez, 2012; Heggie, 2010; Pieper, 2016a, 2016b; Ritchie, 2003; Sullivan, 2011). She passed, received her “femininity certificate,” colloquially called “fem cards” by female athletes, and was able to participate in the championship. Two years later, she won the Spanish University Championship, thereby securing a spot on the 1985 Spanish team for the World University Games in Kobe, Japan.

Martínez-Patiño arrived in Kobe ready to race for a medal. However, because she forgot her “fem card,” she had to again undergo a gender check. “That test in Kobe, which I had taken in Helsinki before, changed my life and my athletic career,” explained the athlete. The doctor informed her that a problem had surfaced and asked that she feigns an injury. “My whole world caved in. It was a nightmare,” she recalled. “They wrapped my ankle to pretend I was hurt, but inside I was destroyed.” This test not only forever changed Martínez-Patiño’s life, but it also changed the direction of gender verification in women’s sport.

When women entered international athletics competitions in the early twentieth century, anxieties surfaced about the acceptability and safety of female participation in the sport (Cahn, 1994; Hargreaves, 1994). Many practitioners worried that track and field was too competitive, too gruelling, and too strenuous for women. Consequently, fears of masculinization and of male imposters rose hand-in-hand with the advancement of female athletes in athletics (Gibb, 1936; Leigh, 1980). To combat issues of supposed unfairness, the IAAF instituted compulsory gender control measures in 1966, and the International Olympic Committee (IOC) followed suit in 1968 (Ferguson-Smith & Ferris, 1991; Hay, 1972; Heggie, 2010). The methods remained largely out of the public spotlight until Martínez-Patiño, with the support of a handful of medical experts, protested the policy.

This article was conceived after the International Academy of Sex Research Presidential symposium in Mexico City in July 2019 “Hurdling over sex? Sports, science, and equity” during which Martinez-Patiño told her story of an elite athlete directly impacted by rules of gender categorization effected by sports authorities. It was further completed by a lengthy interview with Martínez-Patiño, addressing timelines, historical context, gender-based eligibility rules in sports, and gender-related anxieties that have hampered women’s advancement in track and field. It is an important case study for many reasons. First, Martínez-Patiño’s career in athletics illustrates the difficulties women faced when attempting to compete in track and field, both in Spain and internationally. Second, her experience with sex control demonstrates the inadequacies of the chromosomal check, as well as the harms caused by the test. As her experience makes clear, no scientific measure neatly separates women from men for the purpose of sports. Finally, the Spanish runner’s protest of the IAAF’s policy started to dismantle the sex verification policies. The publicity surrounding her case pushed the track and field federation to abandon mandatory, on-site testing in 1992. Seven years later, the IOC also dropped its compulsory control (Genel, 2000; Pieper, 2016a, 2016b). Martínez-Patiño became the face of the fight against sex verification in sport and helped tear down the practice. Yet, her involvement came with a price; she is forever linked to the history of sex testing and gender discrimination.

## Women’s Sport in Franco Spain, 1936–1975

When Martínez-Patiño started competing in track and field, she faced dual obstacles: prevailing gender norms and limited opportunities. “In Spain… there was little support for sport and much less for female sports,” she explained. “It was difficult to see women in the stadiums of track and field, and the level was very low.” Although Spanish women encountered barriers similar to those faced by other European women, the fight for sporting inclusion in Spain presented additional hindrances because of the country’s political strife (Hartmann-Tews & Pfister, 2003). Martínez-Patiño therefore battled encumbering and politicized notions of femininity—as well as a lack of monetary support—to compete at an elite level in track and field.

During the Second Spanish Republic (1931–1939), female sport enjoyed a golden age. The regime not only encouraged women to engage in physical activities, but also extended new rights to them, including suffrage, the prohibition of labor discrimination, and the recognition of divorce. As historian Lopez-Villar (2014) explains, changing attitudes about women changed attitudes about women’s sport. People viewed sport as a way to accentuate female social and political participation. Yet, this golden age proved fleeting as the Spanish Civil War (1936–1939) quashed the brief opening of opportunity.

Under Franco, the dictatorship (1939–1975) entrusted the women’s group with promoting female health and physical education. The regime may have proved less interested in explicitly using sport for overt political gain than its Nazi Germany and fascist Italy counterparts; yet, Franco nevertheless recognized the power of competitions as a domestic recruiting tool. He therefore embraced physical activity for men as a way to create a positive image of the state but he simultaneously opposed “any attempt to extend more modernist notions of the human body to women” (Ofer, 2006).

As a result, female sport under Franco developed in accordance with prevailing gender norms. Dominant ideology demanded that women’s physical activity highlight femininity and serve to create strong offspring (Puig & Soler, 2003). The women’s branch of the Falange (central pillar of Franco’s regime) therefore applauded “beauty sports,” those that preserved appropriate femininity and encouraged women’s participation in basketball, folk dancing, hockey, swimming, and team handball. The Falange discouraged women’s engagement in non-feminine activities, such as boxing, cycling, and track and field (Puig & Soler, 2003). Indeed, writes Ofer (2006), between 1940 and 1963 the Franco dictatorship even deemed female track and field illegal because “in the eyes of many [it] created «mannish women».”

Franco’s death in 1975 marked a turning point for both Spanish politics and Spanish sports. Perhaps most significantly, the country started to slowly move toward a more democratic society. And with these governmental changes came changes in sport, namely the creation of the *Consejo Superior del Deporte* or National Sports Council (by Decree 2258/1977). It replaced the *Delegación Nacional del Deporte*, a body dependent on the *Secretaría General del Movimiento* with ministerial rank, which was mainly devoted to controlling the existing single party and submitting it to Franco’s authority, with the consequent political stability that this produced.

This radical change in sports policy meant an incremental increase in the amount of physical activities available for women and an important shift in the role occupied by women in Spanish sports. Although gender restrictions eventually dwindled, notions of appropriate womanhood continued to limit Spanish women’s advancement in track and field. It was in this environment that Martínez-Patiño started training and was coached as a high-level athlete.

## Martínez-Patiño and Spanish Athletics, 1975–1983

Martínez-Patiño began running in 1976 when she was 13 years old. A coach visited her high school and asked if anyone wanted to join the track and field team. Without much thought, she raised her hand. “That gesture, apparently meaningless, cost me years and years of efforts, struggles, dedication, and overcoming adversities in and out of the stadium,” remembered the Spanish athlete. In raising her hand, she started down a path that forced her to combat hindering gender norms, subpar sporting structures, and gender testing.

Following Franco’s death, Spain underwent a dramatic political transformation. The *Spanish Constitution* of 1978 restored democracy to the country. Spanish sport thereby developed along two main lines: the public sector and the voluntary sector. The public sector consisted of governmentally organized sport entities, such as the autonomous community bodies, city and town councils, and the *Ministry of Education, Culture and Sport*. This sector aimed to make sport accessible for the entire population. The voluntary sector, on the other hand, consisted of private organizations, including professional leagues, Spanish sport federations, the Spanish Olympic Committee, and sport clubs. It was within this realm that elite athletes trained (Puig & Soler, 2003).

Although sport developed in tandem with the democratization of the country, the impact of Franco’s ideals on women’s sport continued after his death. Nevertheless, Spanish sport practitioners combined men’s and women’s sporting programs throughout the 1970s and 1980s. This arrangement intended to assist with athletic equality; however, prevailing gender norms created “serious obstacles” for both female athletes and women’s sport (Puig & Soler, 2003). Most significantly, the expectation that women should prioritize wifely and motherly duties remained intact, as did the concerns with female athletes’ participation in masculine activities.

When Martínez-Patiño raised her hand to join the track and field team in 1976, Spanish athletics was still an under-developed field, and some people still worried about the acceptability of women in the sport. Women’s athletics continued to face opposition because “there was a closed-mindedness” that persisted from “the many years of the dictatorship of General Franco,” explained the runner. These lingering norms shaped both local and national opportunities. Martínez-Patiño felt these pressures first-hand. In her hometown of Galicia, an autonomous community of Northwestern Spain, domestic expectations threatened to hinder her athleticism. She recalled that girls learned to cook, embroider, and sew in order to acquire the skills necessary to run a household. In other words, women were “guided to what would be their future or final destination. [To] be a mother, take care of children and their spouses.” “That was the mentality of that period.” Not surprisingly, these gendered expectations negatively impacted female sport participation. As Gambau (2002) reported, Galician women accounted for only a small percentage of sport participation in the community in the 1970s and 1980s.

Along with preventative gender norms, parental concerns also stopped some girls from engaging in physical activities. For example, before Martínez-Patiño could lace up her spikes, she had to convince her parents to allow her to race. While her father proved supportive, her mother had reservations. “My mother… was opposed and believed that I should not do any sport; but, rather, be educated to be a woman of the house,” she said. Her strong determination to run—her perseverance eventually convinced her mother—was her “first revolution” of many.

With her mother’s reluctant blessing, she took to the track and continued to overcome obstacles. “From the first, I had to go against the system, the costumes, and antiquated ideas of the period,” she explained (Martinez-Patiño, 2005). Her experience was not unusual for the time. Spanish women had to fight for inclusion, equality, and acceptance in sport throughout the 1970s and 1980s. Female athletes received less financial support, substandard equipment and facilities, poorly qualified coaches, and minimal media coverage, trends that still continue today (Puig & Soler, 2003). Additionally, because of girls’ and women’s only recent involvement in sport, few practitioners knew how to properly train female competitors. The Spanish hurdler recalled that her workouts were “not very hard and not very intense,” which resulted in slower times. Finally, in sports considered masculine by the larger society, like track and field, women athletes faced even greater opposition. “In these cases,” write Puig and Soler (2003), “the difficulties and rejection that women encounter[ed] [were] bigger still.” The belief that athletics produced mannish women thus limited the development of track and field in Spain.

Despite the lack of proper coaching, our protagonist remained dedicated. Her life changed dramatically in 1980 when she moved to Madrid to live in the well-known Joaquín Blume Sports Center and train in an elite-level facility. She was the first female scholarship recipient and the only woman in the residence. “Sports was a man’s world,” she surmised. As the lone female athlete on campus, she shouldered the additional burden of serving as the sole representative of her sex. “I thought that it all depended on me, my work and my struggles,” she explained. This motivated her to train harder. Due to her devotion and perseverance, she became one of the fastest women in Spain, dominating the 100-m hurdles. At the 1983 World Championships in Athletics, she finished the event in 13.78 s, earning her a position on the Spanish team headed to Los Angeles for the 1984 Olympic Games and to Kobe for the 1985 World University Games. It was in Helsinki where she first underwent gender control without problems and received her ‘fem card’, but it was in Kobe where issues surfaced.

## The International Association of Athletics Federations’ Sex Testing and Gender Control

After crossing the finish line in 13.71 s, Martínez-Patiño started to prepare for the 1984 Los Angeles Olympic Games and 1985 World University Games. Although the time placed her among the best of the Spanish runners, it paled when compared to her US and European competitors who benefited from better training and greater financial backing. The lack of support in Spain for women’s sport could be seen in the composition of the national team: only three women comprised the female side. “We were vastly outnumbered by so many men,” the athlete said. The notable disparity stemmed from the country’s lack of monetary assistance, substandard coaching, and hindering gender ideals. Restrictive ideals proved acute in Spanish sport; however, similar limitations curved women’s physical abilities internationally. Indeed, the belief that track and field masculinized women persisted in numerous countries (Hartmann-Tews & Pfister, 2003). To reduce this supposed issue and eliminate potential unfair advantages, track and field officials were the first to implement gender control. The IAAF first introduced compulsory testing in 1966 and continued the practice for almost three decades. The IOC followed the IAAF’s lead and introduced testing at all Olympic Games starting in 1968 (Heggie, 2010; Pieper, 2016a, 2016b). Thus, following the IAAF’s regulations, officials at the 1983 World Championships, 1984 Los Angeles Olympic Games*,* and the 1985 World University Games required all female competitors undergo a gender check.

As women started to advance in athletics, the IAAF grew increasingly concerned with the appearances of muscular women (Cahn, 1994; Pieper, 2016a, 2016b; Ritchie, 2003). To prevent male masqueraders from surreptitiously entering female-only events, track and field officials requested athletes produce a medical certificate in 1946, signed by a “qualified medical doctor’, that verified competitors” sex (Heggie, 2010). The IAAF switched tactics 20 years later because of the fear that participants could use fraudulent documents. Therefore, in 1966, the federation mandated an on-site anatomical exam for all female athletes (Pain, 1967). “Sport had no other means of asserting the gender of participants other than having them parade naked in front of a panel of doctors,” reported Ljungqvist (2011), an IAAF member in the 1970s. In the 1966 Asian Games, British Empire and Commonwealth Games, European Athletics Championship, and 1967 Pan American Games, doctors visually inspected female athletes’ anatomy (Pieper, 2016a, 2016b). The humiliating nature of the “nude parade,” combined with the IAAF’s desire for a more scientifically advanced technique, convinced the federation to embrace the Barr body test. Starting in the 1967 European Cup Track and Field Event in Kiev, Soviet Union, the IAAF used the Barr body test to identify chromosomal composition and bar competitors who possessed anything other than an XX constitution. Although problems surfaced immediately, and scientists repeatedly warned that humans did not always fit neatly into XX or XY boxes (Priyadharscini & Sabarinath, 2013), the IAAF continued to require the control.

Because of the IAAF’s steadfast reliance on the Barr body test, Martínez-Patiño and her two teammates had to undergo the check in Helsinki for the 1983 World Championships in Athletics. “We considered it usual for such a test to take place,” she remembered. “It was just something we had to go through.” When the athlete went to the medical center for her exam, her two teammates accompanied her, although they both already possessed “fem cards.” They showed the certificates to the practitioners and were thus considered verified. Her teammates also told Martínez-Patiño not to worry; the test simply confirmed what everyone already knew: she was a woman. She had grown up female and never doubted this fact. For that reason, she was not afraid of providing cheek cells and a sample of her saliva without concern. “I was not, at all, worried,” said Martínez-Patiño, and she did not think twice about the exam. She passed and the IAAF added her name to the international list of verified female competitors. As a verified woman, raced in the 100-m hurdle qualifiers and quarter-finals of the 1983 World Championships in Athletics.

Martínez-Patiño continued to lower her time in the 100-m hurdles in other competitions in 1983. The following year, though, she experienced a great tragedy. Her only brother was diagnosed with leukemia. In response to this situation, she donated bone marrow several times throughout the year in an attempt to save his life. Her efforts were futile. He died that year. Not only did she suffer through the heartbreaking loss of her brother, but the bone marrow donations made her anemic and unable to properly train. As a consequence of this, she missed qualifying for the 1984 Los Angeles Olympic Games by four tenths of a second. “My great dream as well as my great sadness was that I was not able to compete in the Olympics,” she explained. “But I did everything I could to save my brother’s life.”

After enduring such heartache, Martínez-Patiño viewed the 1985 World University Games in Kobe, Japan, with great anticipation. “I was very excited about the championship because the year before I was not able to participate in the Olympic Games*,”* she recalled and arrived in Japan ready to earn the accolades that she had just narrowly missed. First, though, she had to again verify her sex by showing organizers her “fem card.” Although the athlete and her teammates viewed gender testing as a standard procedure, they had accidently left their certificates at home with the RFEA. Thus, the officials required they again undergo the control. Although a bit of a nuisance, the runner did not give it much thought as she had already undergone testing with no problems. She did not know it then, but “that test in Kobe, which I had taken in Helsinki before, changed my life and my athletic career.”

The four Spanish athletes travelled nonchalantly to the medical center on bicycles, entirely unconcerned about their upcoming sex tests. As Martínez-Patiño noted, the control was commonly required in major competitions. They thus went through the standard procedure unperturbed. Everything changed the next day. The Spanish team doctor told the athlete that she—and she alone—had to repeat the check. This time, the exam was more thorough and included a blood sample. Later, the doctors told her she could not compete. “They didn’t give me any details or the opportunity for a secondary test to show that they were mistaken,” she said. “I didn’t understand what was happening or what to do.” The Spanish team doctor told her to feign an injury and skip the race. “I wanted to compete,” she explained, “but they wouldn’t let me.” Instead, with her ankle wrapped in bandages, she watched her competitors run. “The worst moment was to watch the hurdle competition, where I was supposed to compete,” she recalled. “I saw my rivals at the starting blocks ready to run, and I was in the bleacher watching them. Nobody could help me, and I felt so alone.”

Martínez-Patiño’s exclusion not only highlights the unfairness with the method of sex determination in sport, but it also demonstrates the unevenness in its applicability, seemingly based on geographical ties and financial resources. At the 1985 World University Games, US swimmer Kirsten Wengler also had a problem with the test. “I remember sitting in a big room with my teammates. When they handed out the cards, they did not give me one,” Wengler said. “Initially, I thought it was a joke” (Wengler, 2014). A retest indicated the presence of a Y chromatin. Unlike Martínez-Patiño, however, Wengler was allowed to compete. One account posits that she participated because organizers did not have the capability of performing a gynecological inspection, the next step in the procedure for those who “fail.” But Wengler believes her inclusion was because of her appearance. “Visually, I did not look masculine,” she said. “I did not have the appearance of a male so they let me swim” (Wengler, 2014). It is possible that her position as a white, US woman competing in a socially acceptable sport opened doors for her that were not available to others. Moreover, when Wengler returned home, her parents arranged for additional tests that found she was the victim of a false positive.

Martínez-Patiño did not have the same experience. When she returned home after faking an injury, she resumed training. However, the RFEA told her to stop immediately. “They said that I must pretend I am hurt and go home,” said the athlete. Spanish officials suggested she retire discreetly. This appears to be the most common directive given to athletes who “failed” the test. The number of women treated in this fashion remains unknown; however, an Olympic official remarked that this occurred once or twice in every Olympic Games from 1968 to 1988. Most athletes opted to silently retire rather than face international exposure and scrutiny (Hay, 1981).

Martínez-Patiño declined. “I told them that I did not want any more lies and I had all the rights in the world to participate,” she explained. Her refusal to quit resulted in public vitriol and castigation. Yet, her resilience encouraged the medical community’s involvement in a debate about testing and fostered the eventual abandonment of the practice.

## Backlash, Protest, and Change

In January 1986, Martínez-Patiño participated in the Spanish National Games in Oviedo and claimed first place. “I refused to continue the theatrics that the Federation wanted,” she explained. Martínez-Patiño’s victory turned the spotlight on her and unleashed an avalanche of criticism. “I knew that if I participated, the whole world would know what happened in Japan,” she remembered. And word traveled fast. News of her participation and sex test “failure” appeared in headlines around the world. “All the papers in Spain and half of the world had my picture on the cover with the caption, the best woman hurdler has male chromosomes,” said the athlete. “They also mentioned it on the radio and television. It was horrible.” Numerous accounts from around the world exposed her private medical history to a global audience (Mora, 1986). “What happened to me was like being raped,” the athlete recalled in 1991. “I’m sure it’s the same sense of incredible shame and violation. The only difference is that, in my case, the whole world was watching” (Carlson, 1991). As she reeled from the sudden swelling of contempt, the Federation revoked her scholarship, removed her from residence, and erased her records. Martínez-Patiño recognized that she had two options: fight the policy or quietly return to a non-sporting life. She opted to fight.

Not everyone wanted her to quietly quit. Dr Alejandro Doming, Professor at the Universidad Politécnica de Madrid, encouraged her to protest the decision. “He told me that I was a woman and should continue to participate,” she said. He also showed her a *Journal of the American Medical Association* article written by de la Chapelle (1986) that called the IAAF’s sex control measures “inaccurate and discriminatory.” The aforementioned researcher, a Finnish geneticist, had been protesting sex testing for years. He had first written to the IOC Medical Committee in 1982 requesting the group abandon sex testing. This researcher suggested that the Barr body test incorrectly identified and barred intersex women. “When such a woman is found to have an abnormal sex chromatin and excluded from women’s events it constitutes, in my opinion, a flagrant violation of her rights,” he argued. Moreover, de la Chapelle (1982) added that “such an event is likely to create serious, if not fatal, psychological damage.” IOC Member Hay (1983) responded almost a year later and acknowledged that “the procedure is not an absolute safe method” but posited that “it is a practical and economical one.” De la Chapelle (1987b) continued to oppose the practice, writing to various Medical Committee members and IOC President Juan Antonio Samaranch. His efforts initially failed to make change. “I am now quite pessimistic about achieving anything without a public scandal,” he wrote (De la Chapelle, 1987a). Martínez-Patiño’s story provided the spark. With Domingo’s direct support and de la Chapelle’s expertise, she demanded change.

The Spanish hurdler appealed the IAAF’s decision and sued the RFEA. In 1986, a Valencia court ordered the RFEA to pay 20 million pesetas for the “illegal mass dissemination of information and the damage caused by it” (El País, 1986). She also contacted de la Chapelle. “He offered me his scientific help and explained to me, clearly, what was happening to me, thus giving me strength to fight against the injustice committed against me,” she said. Through de la Chapelle, she met with Arne Ljungqvist, IAAF Vice President and IOC Medical Committee member. With Ljungqvist’s assistance, Martínez-Patiño appealed the IAAF’s decision. In 1988, the IAAF reinstated her.

Her story piqued the interest of the medical community. When she stepped forward to protest gender testing, many doctors from around the world stood with her. Numerous endocrinologists, geneticists, and gynecologists believed that the singular use of chromosomes in the determination of sex was scientifically unsound and ethically incorrect (Stephenson, 1996; see also Pieper, 2014, 2016a, 2016b). For example, British geneticist Ferguson-Smith and former British Olympian Elizabeth Ferris similarly argued that the Barr body test erroneously prohibited competitors with androgen insensitivity syndrome, gonadal dysgenesis, Klinefelter syndrome, and mosaicism (Ferguson-Smith & Ferris, 1991). US geneticist Simpson (1986) likewise posited that the singular use of the chromosomal test was inadequate and unfair. The avalanche of criticism launched by Martínez-Patiño eventually convinced the IAAF to abandon compulsory, laboratory-based gender verification in 1992. Seven years later, the IOC followed suit. After two decades of unmediated testing, it was the athlete who sparked the changes in the policy.

Martínez-Patiño’s publicity and protest encouraged the IAAF and IOC to end their compulsory gender verification policies. Although she was pleased to help other women compete unhindered, the accomplishment was not without a cost. “I paid a high price,” she explained. “My story was told, dissected, and discussed in a very public way.” Along with fostering the international scrutiny, the prohibition depleted athletic opportunities of the Spanish runner. “Two years of struggles without training or competing, with a deep pain in my heart, made it very difficult for me to regain my top form,” she said. Martínez-Patiño sat out of the 1988 Olympics. Then, after her reinstatement, she went to the United States and the Soviet Union to train. The hurdler missed qualifying for the 1992 Games by ten hundredths of a second. Thus, her victory in ending testing was bittersweet. “Nobody would have done anything about the injustice if I did not fight it,” she explained. Her sacrifice helped thousands of women. But it came at a significant personal cost.

## Dutee Chand’s Case, Martinez-Patiño’s Witness Statement, and Caster Semenya’s Case

The IAAF offered a rare opportunity to Martínez-Patiño to provide oral testimony in the case of the Indian athlete Dutee Chand. The Athletics Federation of India suspended her from taking part in national and international competitions for the reasons set out under the “IAAF Regulations Governing Eligibility of Females with Hyperandrogenism to Compete in Women’s Competition.” Chand lodged an appeal with the Court of Arbitration for Sport (CAS) against the Athletics Federation of India and the International Association of Athletics Federations (IAAF) (Ospina Betancurt et al., 2018).

The case of the Indian athlete was broadly similar to that of the Spanish athlete. Therefore, Martínez-Patiño said she could identify with Chand: “Like me, she was not a prize-winning athlete, she was not a top athlete. Dutee Chand was a very good athlete, but she was not among the best in the world. However, she will go down in history as a human being on the borderline between men and women, as defined by the regulations. She did not tick all the boxes of womanhood. It was similar to my own experience…”.

For Martínez-Patiño, the attendance at CAS did not involve 100% support for IAAF or for Chand. Although she was called by the IAAF to attend, the witness assured that she felt free to narrate her experience as a person affected by this type of regulations, as detailed under paragraph 320 of the Interim Arbitral Award (CAS 2015).

The current regulations “*IAAF Eligibility Regulations for Female Classification*” (IAAF, 2018) are a stricter version of the earlier regulations challenged by Chand in 2015. These regulations are currently being enforced after the South African athlete Caster Semenya also brought proceedings before CAS like Chand. This time around, CAS did not uphold Semenya’s challenge and, although it found that the Regulations were discriminatory, it deemed that this discrimination was necessary and proportionate for elite female athletics (CAS 2019). However, the regulations have attracted criticism from the scientific community due to a lack of rigor and scientific basis (Franklin et al., 2018; Sőnksen et al., 2018), and this was the same basis used by the CAS itself in the interim arbitration award of the Indian athlete in 2015.

In Martínez-Patiño’s view, the current regulations are “the result of a tug-of-war between a body and an athlete, which have later had an influence on other sportspeople with hyperandrogenism.”

In the 2015 hearing, Martínez-Patiño expert report brought to light that the difference between levels of testosterone of men and women can lead to a competitive advantage (CAS 2015, p. 321). Additionally, she said that certain aspects of the regulations could be improved, above all in areas like confidentiality, privacy, and education. However, she currently believes that “the regulations are biased and put together on an ad hoc basis for one sportswoman.” In 2015, she explained that testosterone can be a differentiating factor in sports performance between men and women. However, in light of the current regulations, it is inconsistent to believe that testosterone is relevant for specific athletics events (400 m, 400 m hurdles, 800 m, 1500 m, and one mile) and not for all events. Additionally, despite published data collected by IAAF showing that there was no competitive advantage in the 1500 m event and the mile, but an advantage in the hammer throw and the pole vault competitions (Bermon & Garnier, 2017), the latter disciplines (throw and vault) were not subject to any of the restrictions on hyperandrogenic athletes, while the former disciplines (1500 m and mile) were restricted.

This makes it apparent that, in reality, the IAAF is not considering testosterone as a differentiating factor in sports performance and, instead, is shifting the argument that some events have a higher rate of female competitors who carry a Y chromosome and therefore should be restricted. This is, in effect, a back-door return to policies based on sex chromosome as a marker for women’s eligibility, which Martinez-Patiño had fought against successfully.

The three cases mentioned in this article, i.e., Martínez-Patiño, Chand, and Semenya, have a striking similarity: they were litigated in the media. The first athlete managed to prove that an error had been committed in applying the regulations in her time, as reflected in the sections above.

Chand was able to return to the tracks and national and international competitions following the interim arbitration award and, finally, the IAAF’s change of criteria. She continues to be a hyperandrogenic athlete, but there are no restrictions in the events of her specialty (100 m and 200 m).

Semenya has not been able to convince the Court that higher levels of testosterone were not solely providing an unfair advantage but has now become a *cause célèbre* for women’s discrimination in sports and in society at-large.

Finally, according to Martínez-Patiño “after the results of the last world championship in Doha 2019, it is unfair to say that Caster Semenya’s superiority is solely due to a hyperproduction of testosterone, when in other cases the superiority and dominance of some female athletes is attributed to natural talent and they are praised for their genetic makeup.” She considers that this rhetoric is unacceptable and is probably in error as far as the regulations are concerned, and the outcome will be very similar to the one provided in her time, i.e., side-lining certain sportswomen, whose greatest mistake was being born and running at the wrong time in history.

## Conclusion

Martínez-Patiño retired from sport on her own volition after she missed the 1992 Olympics. Her love of sport and her desire to help women did not cease, however. She earned a Ph.D. in sport science and now teaches at the University of Vigo. Significantly, she continues to advise the IAAF and IOC on matters related to sex verification in sport. “I never thought that a woman like me, who has gone through so much and has been questioned by the whole world, could now be one of the experts in a group striving to do things differently so that they can be more just,” she said. As the two sport organizations continue to navigate questions of sex and gender, as can be seen from the case of the South African athlete Caster Semenya or previously Foekje Dillema, this athlete aims to ensure women are treated justly and with dignity. “It is important to strive to protect women from the terrible bullying from the media, from the criticism and comments from rivals or administrators,” she explains whenever asked. In other words, she protects them from the mistreatment that she experienced in 1986.

In her role as an advisor to the Medical and Scientific Commission’s of the IOC, Martínez-Patiño strives for fairness for all. She recognizes that making decisions regarding sex and gender in world sports is complex and replete with controversy. While the Spanish runner is opposed to a systematic control of all women, she believes it is important that any decisions made in the future regarding classification and testing consider fair play for everyone and incorporate advanced scientific research. Importantly, Martínez-Patiño listens to all women to “make sure that nobody feels that the regulations are obstacles that prevent them from developing as athletes and human beings without being stigmatized.”

When 13 year-old Maria José Martínez-Patiño nonchalantly raised her hand to join the track and field team, she had no idea she would have such an important influence on the sport and its policies. The trauma she experienced, and her willingness to fight back, have allowed other women to avoid such mistreatment. “My struggles have forced people to think deeply as to the manner in which the tests are to be conducted and that things must be improved,” she explained. She is a pioneer who helped end genetic sex verification. “I came to be part of the history of track and field around the world, of the history of the sport,” she said. “But, that I wouldn’t have known when I raised my hand.”

A case like the one described here cannot be left in our collective memory. National and international sporting institutions must urgently resolve one of the most retrograde scourges of modern sport despite the progress that is being made in the empowerment of women and the recovery of their rights. Sport cannot remain unmoved and respond so slowly and uncertainly in a world of fast-moving social transformations. Scientific advances must be used to support the search for a forceful and effective response to prevent situations of injustice that degrade the dignity of women athletes.

## Data Availability

Not applicable.

## References

[CR1] Bermon S, Garnier PY (2017). Serum androgen levels and their relation to performance in track and field: Mass spectrometry results from 2127 observations in male and female elite athletes. British Journal of Sports Medicine.

[CR2] Cahn SK (1994). Coming on strong: Gender and sexuality in twentieth-century women’s sport.

[CR3] Carlson A (1991). When is a woman not a woman?. Women’s Sport and Fitness.

[CR4] Court of Arbitration for Sports-CAS. (2015). *Dutee Chand v. Athletics Federation of India (AFI) &The International Association of Athletics Federations (IAAF), CAS 2014/A/3759*. Retrieved from http://www.tas-cas.org/fileadmin/user_upload/award_internet.pdf

[CR5] Court of Arbitration for Sports-CAS. (2019). *Caster Semenya, Athletics South Africa (ASA) and International Association of Athletics Federations (IAAF): Decision, CAS 2018/O/5794*. Retrieved from https://www.tas-cas.org/fileadmin/user_upload/Media_Release_Semenya_ASA_IAAF_decision.pdf

[CR6] De la Chapelle, A. (1982, August 17). *Letter to de Alexandre de Mérode*. Commission Medicale correspondance, Avril 1983. Olympic Studies Centre, Lausanne, Switzerland.

[CR7] De la Chapelle A (1986). The use and misuse of sex chromatin screening for “gender identification” of female athletes. Journal of the American Medical Association.

[CR8] De la Chapelle, A. (1987a, April 14). *Letter to Martin Borbrow. Copies of correspondence between professor Albert de la Chapelle and Dr. Martin Bobrow regarding challenges to buccal smear test*. Papers of Malcolm Andrew Ferguson-Smith, University of Glasgow, United Kingdom.

[CR9] De la Chapelle, A. (1987b, May 7). *Letter to Juan Antonio Samaranch*. Commission Medicale correspondance, Mai-Aout 1987. Olympic Studies Centre, Lausanne, Switzerland

[CR32] *El País*. (1986, July 2). *La federación deberá indemnizar a María José Martínez Patiño con 20 millones*. Retrieved from http://elpais.com/diario/1986/07/02/deportes/520639204_850215.html

[CR10] Ferez S (2012). From women’s exclusion to gender institution: A brief history of the sexual categorisation process within sport. International Journal of the History of Sport.

[CR11] Ferguson-Smith MA, Ferris E (1991). Gender verification in sport: The need for change?. British Journal of Sports Medicine.

[CR12] Franklin S, Ospina-Betancurt J, Camporesi S (2018). What statistical data of observational performance can tell us and what they cannot: The case of Dutee Chand v. AFI & IAAF. British Journal of Sports Medicine.

[CR13] Gambau, V. (2002). *Estudio de la Organizacion de los Clubes*. Universidade da Coruna dissertation. Retrieved from https://core.ac.uk/download/pdf/61897839.pdf.

[CR14] Genel M (2000). Gender verification no more?. Medscape Women’s Health.

[CR15] Gibb, A. (1936, May 30). No man’s land of sport. *Toronto Daily Star*, p. 13.

[CR16] Hargreaves J (1994). Sporting females: Critical issues in the history and sociology of women’s sports.

[CR17] Hartmann-Tews I, Pfister G (2003). Sport and women: Social issues in international perspective.

[CR19] Hay E (1972). Sex determination in putative female athletes. Journal of the American Medical Association.

[CR20] Hay, E. (1981, February 22). *Letter to Elizabeth Ferris*. Correspondence between Ferguson-Smith and Dr. Elizabeth A. Ferris on proposed joint article on chromosomal abnormalities and sex test of femininity. Papers of Malcolm Andrew Ferguson-Smith, University of Glasgow, United Kingdom.

[CR18] Hay, E. (1983, April 25). *Letter to Albert de la Chapelle*. Commission Medicale correspondance, Avril 1983. Olympic Studies Centre, Lausanne, Switzerland

[CR21] Heggie V (2010). Testing sex and gender in sports; reinventing, reimagining and reconstructing histories. Endeavour.

[CR22] International Association of Athletics Federations. (2018). *IAAF introduces new eligibility regulations for female classification*. Retrieved from https://www.iaaf.org/news/press-release/eligibility-regulations-for-female-classifica

[CR23] Leigh MH (1980). The enigma of Avery Brundage. Arena Review.

[CR24] Ljungqvist A (2011). Doping’s nemesis.

[CR25] Lopez-Villar C (2014). The beginnings of hockey in 1930s Galicia (Spain): A female phenomenon. International Journal of the History of Sport.

[CR26] Martinez-Patiño MJ (2005). Personal account: A woman tried and tested. Lancet.

[CR27] Mora, J. (1986, January 30). *María José Martínez Patiño estudia demandar a la federación por la forma en que reveló su intimidad*. El País. Retrieved from https://elpais.com/diario/1986/01/30/deportes/507423609_850215.html

[CR29] Ofer I (2006). Am I that body? Sección Femenina de la fet and the struggle for the institution of physical education and competitive sports for women in Franco’s Spain. Journal of Social History.

[CR30] Ospina Betancurt J, Zakynthinaki MS, Martínez-Patiño MJ, CordenteMartinez C, Rodríguez Fernández C (2018). Sex-differences in elite performance track and field competition from 1983 to 2015. Journal of Sports Sciences.

[CR31] Pain, D. (1967, December 14). *Letter to Johann Westerhoff*. IAAF correspondence, 1967–1975, Olympic Studies Centre, Lausanne, Switzerland.

[CR33] Pieper LP (2014). Sex testing and the maintenance of western femininity in international sport. International Journal of the History of Sport.

[CR34] Pieper LP (2016). ‘Preserving la difference’: The elusiveness of sex-segregated sport. Sport in Society.

[CR35] Pieper LP (2016). Sex testing: Gender policing in women’s sport.

[CR36] Priyadharscini RA, Sabarinath T (2013). Barr bodies in sex determination. Journal of Forensic Dental Sciences.

[CR37] Puig N, Soler S, Hartmann-Tews I, Pfister G (2003). Women and sport in Spain. Sport and women: Social issues in international perspective.

[CR38] Ritchie I (2003). Sex tested, gender verified: Controlling female sexuality in the age of containment. Sport History Review.

[CR40] Simpson JL (1986). Gender testing in the Olympics. Journal of the American Medical Association.

[CR41] Sőnksen PH, Bavington LD, Boehning T (2018). Hyperandrogenism controversy in elite women’s sport: An examination and critique of recent evidence. British Journal of Sports Medicine.

[CR42] Stephenson J (1996). “Female Olympians” sex tests outmoded. Journal of the American Medical Association.

[CR43] Sullivan C (2011). Gender verification and gender policies in elite sport: Eligibility and “fair play”. Journal of Sport & Social Issues.

[CR44] Wengler, K. (2014). Phone interview by Lindsay Parks Pieper.

